# Osteocytes regulate bone anabolic response to mechanical loading in male mice via activation of integrin α5

**DOI:** 10.1038/s41413-022-00222-z

**Published:** 2022-07-18

**Authors:** Dezhi Zhao, Rui Hua, Manuel A. Riquelme, Hongyun Cheng, Teja Guda, Huiyun Xu, Sumin Gu, Jean X. Jiang

**Affiliations:** 1 Department of Biochemistry and Structural Biology, University of Texas Health Science Center, San Antonio, TX USA; 2 School of Life Sciences, Northwestern Polytechnical University, Xi’an, China; 3 Department of Biomedical Engineering and Chemical Engineering, University of Texas, San Antonio, TX USA

**Keywords:** Bone, Bone quality and biomechanics

## Abstract

Physical mechanical stimulation can maintain and even increase bone mass. Here, we report an important role of osteocytic integrin α5 in regulating the anabolic response of bone to mechanical loading using an *Itga5* conditional gene knockout (cKO) mouse model. Integrin α5 gene deletion increased apoptotic osteocytes and reduced cortical anabolic responses to tibial compression including decreased endosteal osteoblasts and bone formation, and increased endosteal osteoclasts and bone resorption, contributing to the decreased bone area fraction and biomechanical properties, leading to an enlarged bone marrow area in cKO mice. Similar disruption of anabolic responses to mechanical loading was also detected in cKO trabecular bone. Moreover, integrin α5 deficiency impeded load-induced Cx43 hemichannel opening, and production and release of PGE2, an anabolic factor, resulting in attenuated effects of the loading on catabolic sclerostin (SOST) reduction and anabolic β-catenin increase. Together, this study shows an indispensable role of integrin α5 in osteocytes in the anabolic action of mechanical loading on skeletal tissue through activation of hemichannels and PGE2-evoked gene expression. Integrin α5 could act as a potential new therapeutic target for bone loss, especially in the elderly population with impeded mechanical sensitivity.

## Introduction

The mechanosensitive bone tissue adjusts its architecture to mechanical loading. For example, mechanical loading introduced by physical exercise will increase bone mass, even following a long cessation of stimuli.^1,2^ Conversely, mechanical unloading, such as during space flight and sedentariness, will reduce bone mass.^3,4^ Osteocytes, surrounded by fluid-filled lacunae, are the most abundant embedded in the bone tissue^5^ and connect with neighboring osteocytes and other cells with dendritic processes.^6^ This special location and cell characteristics make osteocytes a major mechanoreceptor in the bone tissue.^7^ Osteocytes can detect the mechanical signals from the lacunar–canalicular network, and release bone anabolic molecules into the bone matrix to modulate bone remodeling on the bone surface.^6,8^

Integrins, consisting of α and β subunits, work in signal transduction processes between cells and extracellular matrix.^9^ Among integrin family members, integrin α5, which only pairs with β1 subunit to form α5 β1 heterodimer, is expressed on the osteocyte surface^10^ and is mechanosensitive.^11^ Integrin α5 is shown to regulate connexin 43 (Cx43) expression^12^ and co-assemble with Cx43 on the plasma membrane by an adaptor protein 14-3-3θ to form the mechanosensitive Cx43 hemichannel complex in osteocytes.^13^ Cx43 hemichannel mediates the exchange of molecules smaller than 1 kDa between the osteocytes and their lacunar–canalicular extracellular microenvironment.^14^ Under fluid flow shear stress (FFSS), integrin α5 interacts directly with Cx43 to open Cx43 hemichannels on osteocytes.^15^ Additionally, the activation of PI3K/AKT by FFSS directly phosphorylates Cx43 and increases their interactions.^16,17^ The opened Cx43 hemichannels release small anabolic factors, such as prostaglandin E2 (PGE2) from osteocytes,^14,18,19^ which functions in an autocrine/paracrine manner^19^ and promotes the bone anabolic response to mechanical loading.^20^ Based on the interaction between integrin α5 and Cx43 hemichannels, we hypothesize that integrin α5 promotes the anabolic function of mechanical stimulation in bone through the activation of Cx43 hemichannels.

Homozygous integrin α5 null mice die due to severe posterior and extraembryonic mesodermal defects.^21^ To determine the roles of integrin a5 in osteocytes and its anabolic function during mechanical loading, we deleted the integrin a5 gene in osteocytes using the 10-kb dentin matrix protein 1 (*Dmp1*) promoter-driven Cre. In this study, we showed that integrin α5 deletion in osteocytes impeded anabolic responses to mechanical loading in cortical and trabecular bones. We further unveiled the importance of integrin α5 in the activation of osteocytic hemichannels and PGE2 release in response to bone mechanical loading and the underlying mechanism regarding the role of osteocytic integrin α5 in load-induced bone remodeling.

## Results

### Deletion of integrin α5 in osteocytes increased osteocyte apoptosis in cortical bone

**Fig. 1:**
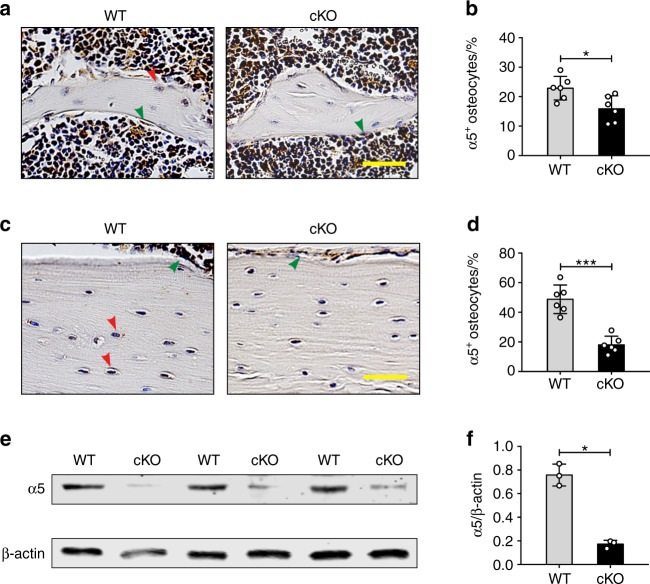
Deletion of integrin α5 gene in osteocytes, but not in osteoblasts in cKO mice. Representative images of integrin α5 immunohistostaining in tibial metaphyseal trabecular bone (**a**) and midshaft cortical bone (**c**) of WT and cKO mice. The red arrow shows α5-positive osteocytes and the green arrow shows α5-positive osteoblasts. (**a**, **c**) Scale bar: 40 μm. Quantification of integrin α5-positive osteocytes in tibial trabecular bone (**b**) and cortical bone (**d**) of WT and cKO mice. *n* = 6 per group. **e**, **f** Integrin α5 deletion was confirmed by western blot of marrow-flushed tibia bone extract samples. *n* = 3 per group. Mean ± SD. *P < 0.05; ***P < 0.001. Student unpaired *t* test

It has been shown that osteocyte survival and bone structure in vivo are regulated by Cx43 hemichannels,^22^ and integrin α5β1 is required for Cx43 hemichannel activation during mechanical stress in vitro.^15^ We hypothesized that the removal of osteocytic integrin α5 may reduce hemichannel activity and compromise the anabolic function of bone during mechanical loading. To test this hypothesis, we generated osteocytic integrin α5 cKO mice using the Cre/LoxP system.^23^ Immunohistochemical staining confirmed that integrin α5 was efficiently deleted in osteocytes of tibial metaphyseal trabecular bone (Fig. 1a, b) and midshaft cortical bone in *Dmp1-Cre*; *α5*^*flx/flx*^ (cKO) mice compared to *α5*^*flx/flx*^ (WT) littermates (Fig. 1c, d). As expected, most osteoblasts on the tibial metaphyseal trabecular and midshaft cortical bone surface were integrin α5 positive (green arrowheads), demonstrating the osteocyte-specific deletion of integrin α5 (Fig. 1a, c). Correspondingly, western blots showed that the integrin α5 protein was barely detectable in bone marrow-flushed tibia extracts from cKO mice, in contrast to that in WT littermates (Fig. 1e, f). WT and cKO mice at 1 to 4 month-old did not show much difference in body weight (Fig. S1A), as well as tibial and whole-body bone mineral density (BMD), scanned by dual-energy X-ray absorptiometry (DEXA) (Fig. S1B, C), indicating that the deletion of integrin α5 from osteocytes did not affect bone accrual.

**Fig. 2:**
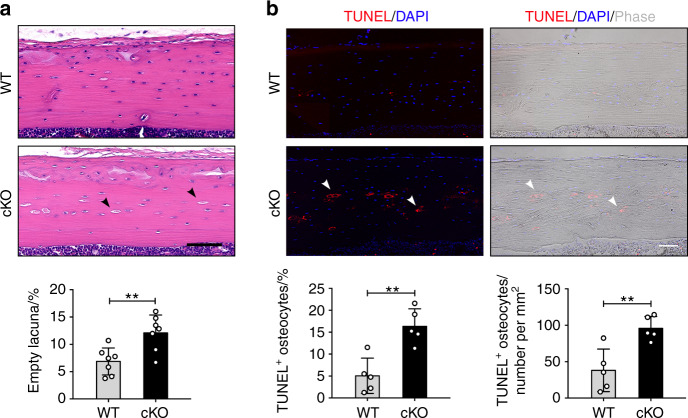
Deletion of integrin α5 in osteocytes increased osteocyte apoptosis and empty lacunae in tibial cortical bone. **a** Representative H&E images and empty lacunae quantification (black arrows) of tibial midshaft cortical bone in WT and cKO mice. Scale bar: 80 μm. *n* = 7 per group. **b** Representative fluorescence images of TUNEL-staining (left upper panels) and corresponding phase images (right upper panels). The TUNEL-positive osteocytes are indicated (white arrows). TUNEL-positive osteocytes were quantified in tibial cortical bone based on total lacunae and bone area (lower panels). Scale bar: 60 μm. *n* = 5 per group. Mean ± SD. **P < 0.01. Student unpaired *t* test

Hematoxylin and eosin (H&E)-stained tissue sections of tibia cortical bone (Fig. 2a, upper panel) and quantitative analysis (Fig. 2a, lower panel) showed more empty lacunae in cortical bones of cKO mice compared to WT mice at 15 weeks of age. Apoptotic osteocytes were confirmed using terminal deoxynucleotidyl transferase dUTP nick end labeling (TUNEL) staining. As shown in midshaft cortical bone, there were increased TUNEL-positive osteocytes (red) in cKO mice compared to WT mice (Fig. 2b, upper panel). The TUNEL labeling validated the significant increase of apoptotic osteocytes in cKO mice compared to WT mice (Fig. 2b, lower panels). Ploton-silver staining showed that cKO mice had shorter and fewer osteocyte dendrites relative to WT mice in the region near the endocortical surface (Fig. S2A, B). However, both cKO and WT mice had similar osteocyte dendrites in the region close to the periosteal surface (Fig. S2C, D).

### Integrin α5 deficiency in osteocytes impeded anabolic responses of tibial bone to mechanical loading

**Fig. 3:**
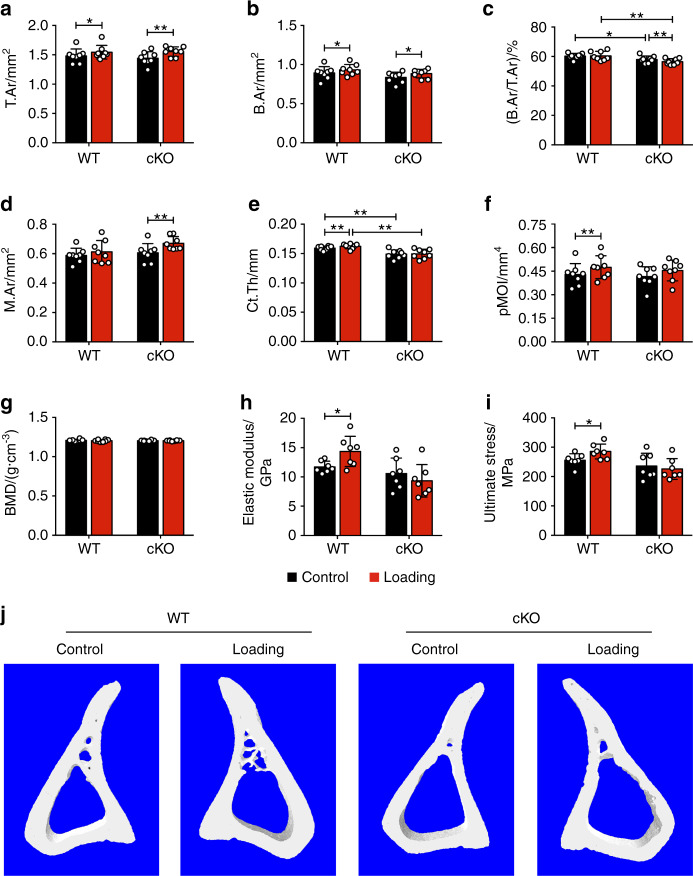
Deletion of integrin α5 in osteocytes attenuated or reversed the anabolic function of mechanical loading in midshaft cortical bone. μCT analysis of the mid-diaphyseal region located 37% from the proximal end, for both loaded and contralateral, unloaded tibias of WT and cKO mice: **a** total cross-sectional area (T.Ar), **b** bone area (B.Ar), **c** bone area fraction (B.Ar/T.Ar), **d** bone marrow area (M.Ar), **e** cortical thickness (Ct.Th), **f** polar moment of inertia (pMOI), and **g** bone mineral density (BMD). *n* = 8 per group. **j** Representative 3D models of the cortical bone for WT and cKO mice. Three-point bending test was performed for tibias bones of WT and cKO mice: **h** elastic modulus and **i** ultimate stress. *n* = 7 per group. Mean ± SD. *P < 0.05; **P < 0.01. Paired *t* test was used for loaded and contralateral tibias and unpaired *t* test for loaded or control tibias between WT and cKO mice

To investigate the in vivo role of integrin α5 in the anabolic responses to mechanical loading, we subjected WT and cKO male mice to a 2-week cyclic axial tibial compression loading. Similar to previous studies,^24,25^ this strain level did not cause potential osteoarthritic damage in medial tibial plateau (MTP) (Fig. S3A, B) and medial femoral condyle (MFC) regions (Fig. S3A, C). We chose the diaphysis located 37% distal from the proximal end to analyze the cortical bone structure (Fig. S4). This volume of interest (VOI) corresponds with previously published studies that show the osteogenic response to axial loading at this location is the greatest.^25,26^ Micro-computed tomography (μCT) analysis showed a significantly lower bone area fraction (B.Ar/T.Ar) and cortical thickness (Ct.Th) in non-loaded contralateral tibias in cKO mice than in WT mice (Fig. 3c, e). Compared to the contralateral control tibias, mechanical loading increased Ct.Th in WT mice (Fig. 3e). Although loading caused increased bone area (B.Ar), as a result of the enlargement of total cross-sectional area (T.Ar) over the contralateral control tibias in cKO mice (Fig. 3a, b), enlarged bone marrow area (M.Ar) (Fig. 3d) and decreased cortical thickness (Fig. 3e) led to a loss of the bone area ratio, as shown by decreased B.Ar/T.Ar in cKO mice (Fig. 3c). As a result, the increased Ct.Th and polar moment of inertia (pMOI) induced by tibial loading shown in WT mice were not observed in cKO mice (Fig. 3e, f). The BMD did not change during mechanical loading in both WT and cKO mice (Fig. 3g). The three-point bending analysis showed significantly increased elastic modulus and ultimate stress versus the contralateral controls in WT mice, but such an increase was not found in cKO mice (Fig. 3h, i). Figure 3j shows the representative 3D images of cortical bone.

In metaphyseal trabecular bone, mechanical loading increased the trabecular thickness (Tb.Th) over their contralateral controls in both cKO and WT mice (Fig. S5A). However, cKO mice exhibited a decrease in trabecular number (Tb.N) and an increase in trabecular separation (Tb.Sp) compared with the contralateral controls in response to tibial loading (Fig. S5B, C). There was no change in bone volume fraction (BV/TV), structural model index (SMI), and BMD in both WT and cKO mice (Fig. S5D–F). Fig. S5G shows the representative 3D images of trabecular bone. Together these results indicate that integrin α5 participates in the anabolic responses of both cortical and trabecular bone to mechanical loading.

### Deletion of integrin α5 from osteocytes inhibited the load-induced increase in midshaft endosteal osteogenesis

**Fig. 4:**
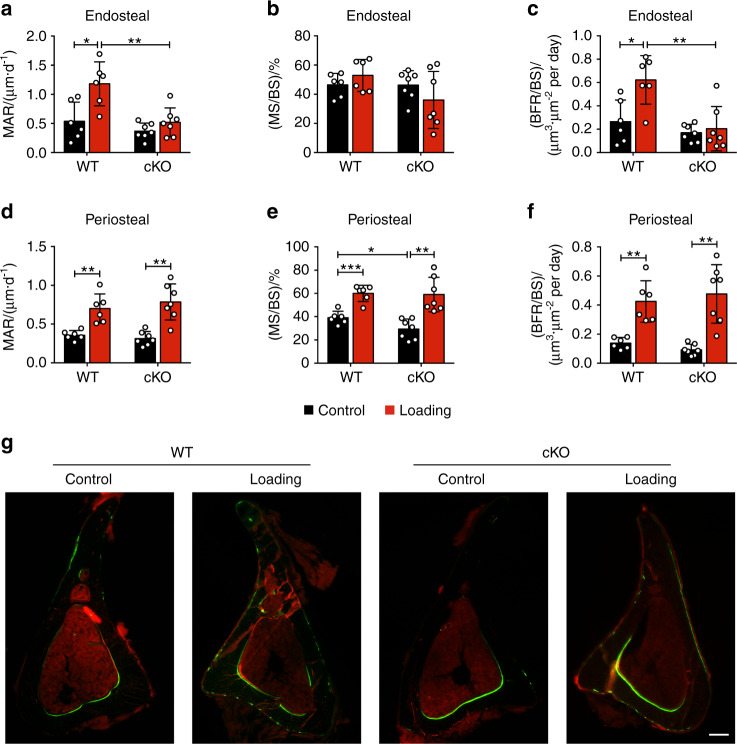
Integrin α5 deletion in osteocytes inhibited increased midshaft endosteal osteogenesis by mechanical load. Bone dynamic histomorphometry was conducted on the tibias within diaphyseal 37% cortical VOI, for both loaded and contralateral, unloaded tibias of WT and cKO mice. Mineral apposition rate (MAR) (**a**, **d**), mineralizing surface/bone surface (MS/BS) (**b**, **e**), and bone formation rate/bone surface (BFR/BS) (**c**, **f**) were assessed along endosteal (**a**–**c**) and periosteal (**d**–**f**) surfaces of all tibias. *n* = 6 per group. **G** Representative images of calcein (green) and alizarin (red) double labeling at the 37% VOI for all groups. Scale bar: 200 μm. *n* = 6 per group. Mean ± SD. *P < 0.05; **P < 0.01; ***P < 0.001. Paired *t* test was performed for loaded and contralateral tibias and unpaired t test was performed for loaded or control tibias between WT and cKO mice

Dynamic histomorphometry of non-loaded contralateral tibias in WT and cKO mice showed that on the endosteal surface, mechanical loading caused an increase in mineral apposition rate (MAR) and bone formation rate/bone surface (BRF/BS) over the contralateral controls in WT, but such increase was inhibited in cKO mice (Fig. 4a, c). Furthermore, MAR and BFR/BS in loaded tibias were greater in WT mice than in cKO mice (Fig. 4a, c). However, no significant increase in mineralizing surface/bone surface (MS/BS) by tibial loading was observed, while a decreasing trend was shown in loaded cKO tibia (Fig. 4b). Representative images showed that the endosteal osteogenic response is concentrated in the postero-lateral region (Fig. 4g), which was also seen in other studies with similar tibial loading models.^24,27^ In contrast to the endosteal surface, MS/BS was significantly decreased in cKO mice compared to WT on the periosteal surface at the diaphyseal 37% region (Fig. 4e). MAR, MS/BS, and BFR/BS were significantly increased on the periosteal surface, similar to their contralateral controls in both cKO and WT mice (Fig. 4d–f). These results show that the deletion of integrin α5 from osteocytes attenuates endosteal bone formation caused by mechanical loading.

### Integrin α5 deficiency inhibited the load-induced increase in osteoblast activity but promoted osteoclast activity in trabecular bone

**Fig. 5:**
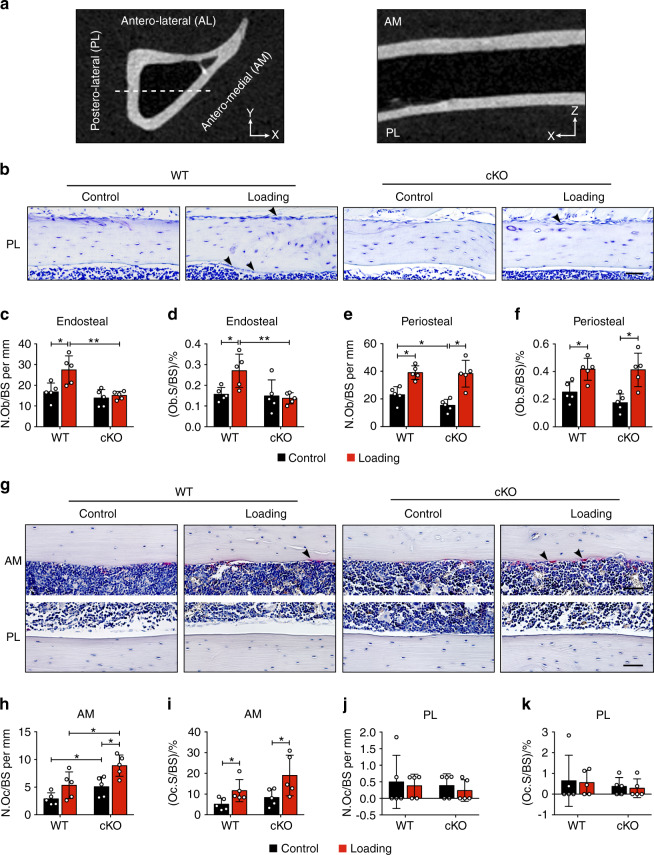
Integrin α5 in osteocytes inhibited loading-induced increase in osteoblast activity. **a** Gray-scale μCT images of transverse, sagittal sections. The dotted lines showed the locations of sagittal paraffin sections on transverse cross-section of the tibia bone (left panel) and the sagittal image showed the paraffin sections obtained (right panel). **b** Representative toluidine blue staining showed the osteoblasts on postero-lateral (PL) periosteal and endosteal surfaces of 37% diaphysis, for both loaded and contralateral, unloaded tibias of WT and cKO mice. The representative osteoblasts are indicated (black arrows). Scale bar: 50 μm. Quantification of osteoblast number per bone perimeter (N.Ob/BS) (**c**, **e**) and osteoblast surface per bone perimeter (Ob.S/BS) (**d**, **f**) on PL endosteal (**c**, **d**) and periosteal (**e**, **f**) surfaces of 37% diaphysis. *n* = 5 per group. **g** Representative images of TRAP-positive osteoclasts (black arrow) on antero-medial (AM) and PL endosteal surfaces of 37% diaphysis, for both loaded and contralateral, unloaded tibias of WT and cKO mice. Scale bar: 50 μm. Quantification of TRAP-positive osteoclast number per bone perimeter (N.Oc/BS) and osteoclast surface per bone perimeter (Oc.S/BS) on AM (**h**, **i**) and PL (**j**, **k**) endosteal surfaces of 37% diaphysis. *n* = 5 per group. Mean ± SD. *P < 0.05; **P < 0.01^.^ Paired *t* test was done for loaded and contralateral tibias and unpaired *t* test was done for loaded or control tibias between WT and cKO mice

The increased bone marrow cavity area of the loaded α5 cKO mice induced by mechanical loading could be a result of unbalanced activities of osteoblasts and osteoclast. Sagittal paraffin sections were used to study the postero-lateral and antero-medial regions, as shown by the gray-scale μCT images (Fig. 5a). Dotted lines indicate the locations of the paraffin sections in the transverse cross-section of the tibia (Fig. 5a, left panel) and the sagittal image showed the paraffin sections obtained (Fig. 5a, right panel). Given the strong endosteal osteogenic response occurring on the postero-lateral (PL) surface (Fig. 4g), we focused on analyzing osteoblasts in this region. Consistent with the dynamic histomorphometry data, histological analysis of contralateral, non-loaded tibias of the WT and cKO mice showed a significant loading-induced increase of osteoblasts over contralateral controls in WT mice, but not in cKO mice on the endosteal surface (Fig. 5b–d). Furthermore, the increase of osteoblasts in loaded tibias was greater in WT mice than in cKO mice (Fig. 5c, d). Moreover, there were fewer osteoblasts on the periosteal surface in cKO mice compared to WT mice (Fig. 5b, e). Load-induced increase of osteoblasts on the periosteal surface is similar in WT and cKO mice (Fig. 5e, f). Since M.Ar was enlarged in cKO mice upon loading (Fig. 3d), we hypothesized that this response was partly due to increased osteoclast activity. As expected, mechanical loading increased the osteoclasts on the antero-medial (AM) endosteal surface over contralateral controls in both WT and cKO mice (Fig. 5g–i). In contrast, osteoclasts on the PL endosteal surface did not change during mechanical loading (Fig. 5j, k). Analysis of contralateral, non-loaded tibias showed more osteoclasts in cKO mice than WT mice (Fig. 5h). Interestingly, mechanical loading significantly increased osteoclast numbers and osteoclast surfaces in metaphyseal trabecular bone in cKO mice (Fig. S6). These data suggest that integrin α5 modulates the osteoblastic and osteoclastic activities during mechanical loading.

### Integrin α5 deficiency ablated the opening of hemichannels induced by mechanical loading in osteocytes

**Fig. 6:**
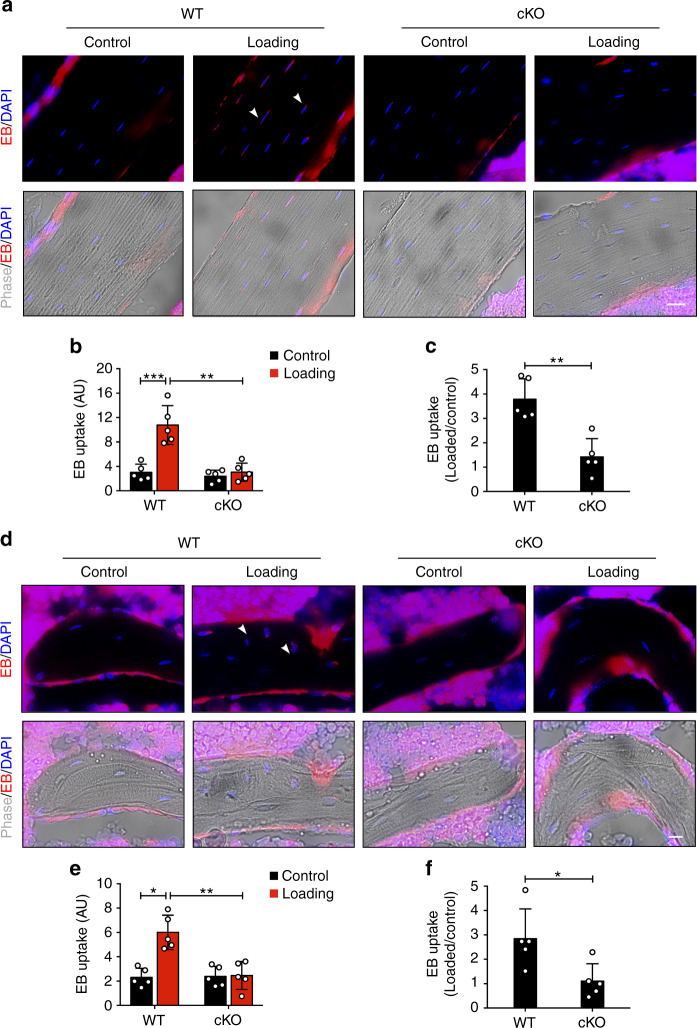
Hemichannel opening in osteocytes induced by mechanical loading was inhibited in integrin α5 cKO. **a** Representative fluorescence images of EB dye uptake in diaphyseal 37% cortical VOI, for both loaded and contralateral, unloaded tibias of WT and cKO mice. The white arrowheads indicated the EB-positive osteocytes. Scale bar, 20 μm. **b** Quantitation of EB fluorescence intensity in osteocytes in cortical bone for both loaded and contralateral, unloaded tibias of WT and cKO mice. **c** EB fluorescence intensity is presented as a fold increase relative to non-loaded groups. *n* = 5 per group. **d** Representative images of EB dye uptake in metaphyseal trabecular bone for both loaded and contralateral, unloaded tibias of WT and cKO mice. The white arrowheads indicated the EB-positive osteocytes. Scale bar, 10 μm. **e** Quantification of EB fluorescence intensity in osteocytes in metaphyseal trabecular bone for both loaded and contralateral, unloaded tibias of WT and cKO mice. **f** EB fluorescence intensity is presented as a fold increase relative to non-loaded groups. *n* = 5 per group. Mean ± SD. *P < 0.05; **P < 0.01; ***P < 0.001. Paired *t* test was performed for loaded and contralateral tibias and unpaired *t* test was performed for loaded or control tibias between WT and cKO mice

Our previous studies have indicated that knocking down integrin α5 in cultured osteocytes abolished fluid flow-induced opening of Cx43 hemichannels.^15^ To determine whether hemichannels were involved in the integrin α5-regulated anabolic response to axial tibial loading, we assessed hemichannel activity in situ in WT and cKO mice by injecting Evans blue (EB) dye into mouse tail veins. Frozen bone sections at the mid-diaphyseal region showed that mechanical loading increased EB uptake of osteocytes in WT mice, but not in cKO mice (Fig. 6a). The quantification of EB fluorescence intensity confirmed the inhibited dye uptake in cKO mice (Fig. 6b, c). Similarly, mechanical loading also increased EB dye uptake in the metaphyseal trabecular bone, but such increase was inhibited in cKO mice (Fig. 6d–f). These results suggest that integrin α5 plays an important role in regulating the opening of osteocytic hemichannels in response to mechanical loading.

### Loss of loading-induced PGE2 increase and sclerostin (SOST) decrease in cKO mice

**Fig. 7:**
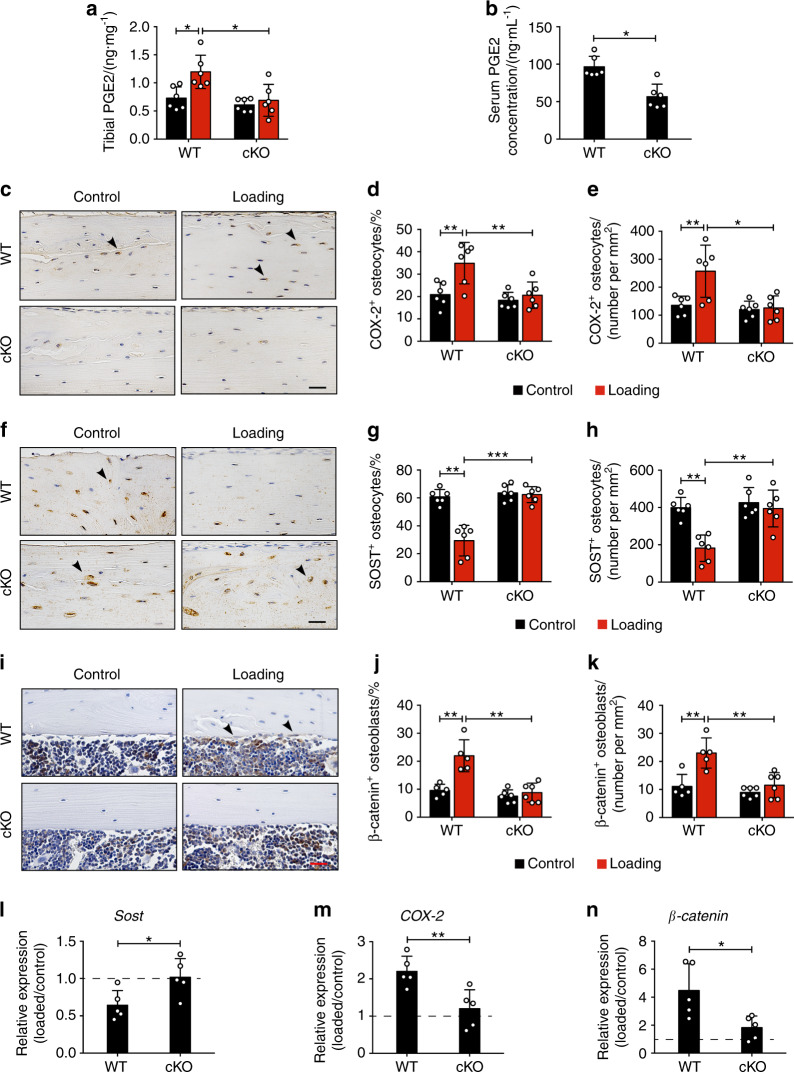
Loading-induced increased PGE2 secretion with a decrease in SOST expression was inhibited in cKO mice. **a**, **b** ELISA analysis of PGE2 level in bone marrow-flushed tibial diaphysis (**a**) and serum (**b**) after 5-day mechanical loading. *n* = 6 per group. **c** Representative COX-2 immunohistostaining (black arrows) and **d**, **e** quantification of COX-2-positive osteocytes in diaphyseal 37% cortical bone. Scale bar, 30 μm. *n* = 6 per group. **f** Representative SOST immunohistostaining (black arrows) and **g**, **h** quantification of SOST-positive osteocytes in diaphyseal 37% cortical bone. Scale bar, 30 μm. *n* = 6 per group. **i** Representative β-catenin immunohistostaining (black arrows) and **j**, **k** quantification of β-catenin-positive osteoblasts on endosteal surface of diaphyseal 37% cortical bone. Scale bar, 30 μm. *n* = 5–6 per group. **l**–**n** Relative gene expression of *Sost* (**l**), *COX-2* (**m**), and *β-catenin* (**n**) was determined by RT-qPCR in the tibial diaphysis of WT and cKO mice. *n* = 5 per group. Mean ± SD. *P < 0.05; **P < 0.01; ***P < 0.001. Paired *t* test was done for loaded and contralateral tibias and unpaired *t* test was done for loaded or control tibias between WT and cKO mice

Extracellular PGE2 released from Cx43 hemichannels^14^ is crucial to the bone mechanical response,^28,29^ thus we detected PGE2 levels in the serum and tibias in both WT and cKO mice. Loading induced a significant increase of PGE2 level over the contralateral controls only in WT mouse tibias, but such increase was impeded in cKO tibias (Fig. 7a). The PGE2 level was also higher in WT than in cKO mice in serum (Fig. 7b). Immunohistochemical analysis also showed that the COX-2, the enzyme responsible for PGE2 synthesis, has a significant increase of the expression in osteocytes over the contralateral controls in tibial cortical bone in WT mice. However, the increased COX-2 expression in loaded tibias was not detected in cKO mice (Fig. 7c–e). We then determined the involvement of integrin α5 in loading-induced SOST suppression. Mechanical loading reduced SOST-positive osteocytes compared to contralateral controls in WT mice, but this decrease in loaded tibias did not occur in cKO mice (Fig. 7f–h). Since the downregulation of SOST in osteocytes is vital for loading-induced WNT pathway activation,^30^ we determined expression of another WNT signaling molecule β-catenin in osteoblasts. Mechanical loading caused significantly increased expression of β-catenin-positive osteoblasts on the endosteal surface, compared to the contralateral control tibias from WT mice; in contrast, the increase was absent in cKO mice (Fig. 7i–k). Relative gene expression by RT-qPCR further confirmed that the decreased *Sost* and increased *COX-2* and *β-catenin* gene expression observed was impeded in cKO mice (Fig. 7l–n). The results indicate that during mechanical loading, deletion of integrin α5 from osteocytes impedes the release of PGE2 and then decreases SOST in osteocytes, which is correlated with impeded β-catenin expression and osteoblast activity on the endosteal surface.

## Discussion

Integrin α5 in osteocytes is highly responsive to mechanical stimulation and its activation leads to Cx43 hemichannel activation in cultured osteocytes;^15,17^ however, its roles in the bone anabolism to mechanical loading are largely unknown. In the current study, using osteocytic integrin cKO mice and an established axial tibia loading model, we demonstrate that integrin α5 is essential to the osteogenic response to tibial loading, and that the response is associated with hemichannel opening and PGE2 release.

There is a significant increase of empty lacunae and osteocyte apoptosis in tibial cortical bone of cKO mice. These findings share certain characteristics with earlier studies in Cx43 transgenic mice with impaired osteocytic Cx43 hemichannels as well as Cx43 cKO in osteocytes, including increased empty lacunae and apoptotic osteocytes in cortical bones compared to control mice.^22,31^ Since Cx43 hemichannels in osteocytes could transduce cell survival signals^32^ and integrin α5 and Cx43 interaction is necessary for the opening of Cx43 hemichannels during mechanical stimulation,^15^ it is in agreement that the deficiency of integrin α5 leads to increased osteocyte death, suggesting that these two proteins may work together in vivo. Osteocyte apoptosis was associated with osteoclast recruitment and localized bone resorption.^33,34^ Consistently, we showed that the increased osteoclasts on the tibial endosteal surface were associated with slightly expanded bone marrow area and reduced bone area fraction. This phenomenon was observed in Cx43 transgenic mice with impaired Cx43 hemichannels in osteocytes.^22^ Interestingly, in contrast to transgenic mice which have increased periosteal bone formation, cKO mice showed lower MS/BS and osteoblast activity on the periosteal surface. This difference could be explained by the other roles of integrin α5, such as mechanosensitive adhesions^35^ in addition to its regulation of Cx43 hemichannels. The study suggests that integrin α5 plays a crucial role in the survival of osteocytes by regulating endosteal bone resorption and periosteal bone formation, and its deficiency results in decreased cortical thickness.

We determined the role of osteocytic integrin α5 using an axial tibial compression model. Our results demonstrated differential responses to mechanical loading occurring on the periosteal and endosteal bone surfaces. Mechanical loading showed a significant increase in bone formation and osteoblast activity on the tibial postero-lateral endosteal surface in WT mice. Postero-lateral endosteal surface is the region where the peak strain occurs, which is 1.5 to 2 times higher than those on the antero-medial face where the strain gauge is placed.^36^ Indeed, cortical modeling and remodeling are more profound on the posterior endosteal surface,^37^ and the mechanical response mainly occurred on the endosteal surface.^38^ However, integrin α5 deficiency attenuated the bone formation response on the endosteal surface. Since dendritic processes of osteocytes are crucial mechanotransducers,^7,15^ decreased osteocyte dendrites near the endosteal surface in cKO mice may compromise mechanical transmission responses, which may lead to impeded endosteal bone formation response to mechanical stimulation. Concurring with this observation, Cx43 knockout in osteoblasts/osteocytes also showed a weak increase in endosteal bone formation,^39,40^ or a greater reduction in endosteal formation in response to tibial loading.^41^ These in vivo evidence indicate a close relationship between integrin α5β1 and Cx43 in the response to mechanical loading in bone tissue. Interestingly, mechanical loading stimulated osteoclast activity on the antero-medial endosteal surface in both WT and α5 cKO groups. The data is consistent with the study by Birkhold et al., showing that bone resorption increased with loading at medial regions in both metaphysis and diaphysis.^37^ Thus, tibial loading promotes endosteal osteoblast activity, which is probably regulated by mechanosensitive integrin α5.

On the contrary, tibial loading cause an increase in periosteal bone formation and total tissue area in both WT and α5 cKO mice. We exclude the regional structural differences because of similar osteocyte dendrites near periosteal surface in WT and cKO mice. In agreement with our findings, Cx43 gene deletion from osteoblastic cells^41,42^ or osteocytes^43^ also showed enhanced periosteal osteogenic response to mechanical loading. These results indicate that osteoblasts on periosteal side are intrinsically more sensitive to mechanical stimulation. It is possible that the remaining integrin α5 in osteocytes may interact with Cx43 hemichannels in the osteocytes close to periosteal surface where the strain is higher than endosteal surface, which may activate another signaling pathway that recuses the reduced mechanical response due to the absence of integrin a5 in osteocytes. On the other hand, previous works showed that angiogenesis was mediated by the mechanical environment of the extracellular matrix.^44^ Mechanical loading stimulates the increase of periosteal angiogenesis at sites of bone formation in mice and rats.^45,46^ From the aspect of mechanical property, increased cortical bone size and bone marrow area in cKO mice are adaptive responses, allowing the bone to endure high levels of stress.^47^ Thus, the osteogenic response of osteocytic integrin α5 to mechanical loading should be separated on endosteal surfaces and periosteal surfaces. Tibial loading accelerates osteoblast recruitment to the endosteal surface, which is likely mediated by mechanosensitive integrin α5. Excessive endosteal osteoclast activity coupled with increased periosteal osteoblast activity led to enlarged bone marrow cavity and attenuated bone area fraction in cKO mice.

Apart from cortical bone, the trabecular anabolic response to mechanical loading was impeded or even reversed in cKO mice, as manifested by decreased trabecular numbers and increased space. Increased osteoclasts on trabecular bone in cKO mice indicated the enhanced bone resorption caused by mechanical loading. A similar load-induced increase of trabecular osteoclasts was found in a previous study using ovariectomized mice.^48^ We have previously shown that the ovariectomized mouse model also had reduced hemichannel function in osteocytes.^49^ Interestingly, the mechanical loading-induced trabecular bone loss did not correlate with the increased trabecular thickness in cKO mice. A previous study also reported an increase in trabecular thickness, but bone loss during aging.^50^ One possibility, as proposed before, is that trabecular bone with lower mechanical sensitivity may disappear due to higher osteoclast activity, while trabecular bone with higher mechanical sensitivity may enhance load-induced anabolic responses by thickening trabecular bone. This was considered as a loss of metastable equilibrium.^51^ Thicker trabecular bone is likely to develop resistance to the reduction of mechanical properties due to trabecular bone loss. A similar load-induced increase of trabecular osteoclasts was found in ovariectomized mice.^48^ Together, these observations indicated that integrin α5 deficiency reduced mechanical sensitivity and accelerated bone remodeling in trabecular bone.

Previous in vitro studies have reported that integrin α5 interacts directly with Cx43 to open Cx43 hemichannels in the osteocyte cell body in response to FFSS,^13,15^ through PI3K/AKT signaling mechanism.^16,17^ We also show that the deletion of integrin α5 in *Dmp1-Cre*; *α5*^*flx/−*^ mice attenuated the mechanical sensitivity of osteocytic Cx43 hemichannels in tibias during mechanical loading.^17^ We showed here that the deletion of integrin α5 impeded the opening of osteocytic hemichannels induced by tibial loading in both trabecular and cortical bone. The opening of Cx43 hemichannels caused by FFSS mediates the PGE2 release.^14,18^ Extracellular PGE2 is a skeletal anabolic modulator that is synthesized and released by osteocytes during mechanical loading (Jee et al.^29^; Thorsen et al.^28^). Mechanical stimulation induces increased PGE2 level in the proximal tibial metaphysis in healthy women.^28^ Intermittent PGE2 treatment increases endosteal bone formation^29^ and bone mass.^52^ Conversely, mice lacking COX-2 attenuates endosteal bone formation during mechanical loading in rat tibia bone.^53^ Here, we found that tibial loading increased both PGE2 levels and its synthetic enzyme COX-2 expression in osteocytes in tibial bone. However, the PGE2 and COX-2 increase were inhibited in cKO mice. The reduced COX-2 expression could be caused by a feedback inhibitory mechanism via its end product, PGE2 due to the blockade of PGE2 release by impaired hemichannels. Similar findings were reported recently in our dominant negative Cx43 mutants driven by the 10-kb *Dmp1* promoter^20^ and previously in Cx43 cKO mice driven by the 8-kb *Dmp1* promoter.^41^ We further showed that the deletion of integrin α5 from osteocytes impaired the opening of hemichannels and inhibited COX-2 and PGE2 release, associated with an attenuation of endosteal anabolic response to mechanical stimulation.

**Fig. 8:**
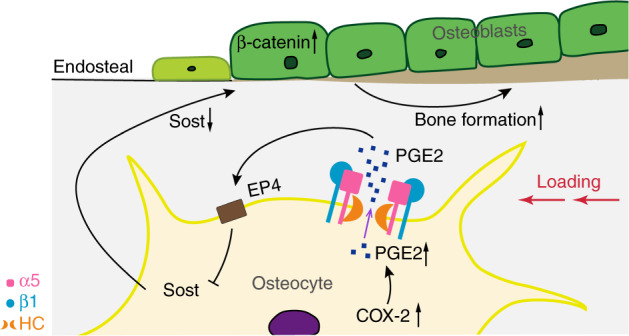
Illustration of the role of osteocytic integrin a5β1 in anabolic response to mechanical stimulation in bone. Upon mechanical loading, COX-2 and PGE2 level are increased. Integrin α5β1 expressed on the osteocyte surface will be activated to change its extended extracellular domain and trigger the opening of Cx43 hemichannels as shown previously.^15^ The opened hemichannels will release PGE2 that functions in an autocrine manner^84^ through the EP4 receptor to reduce SOST expression.^54^ As a result, β-catenin expression is increased in osteoblasts, which leads to an increase in osteoblast activity and endosteal bone formation. HC, hemichannel

Our previous studies reported that the PGE2 release via osteocytic Cx43 hemichannels acts in an autocrine manner via the EP2 receptor in response to FFSS.^19^ The binding of PGE2 to the EP4 receptor reduces the SOST expression,^54^ which acts as an antagonist of Wnt-β-catenin^55^ to inhibit osteogenesis by its binding to the Wnt co-receptor Lrp5/6^56^ and suppressing β-catenin expression in osteoblasts.^57^ The Wnt/β-catenin signaling pathway plays an important role in mechanotransduction. Increased activity of Wnt/β-catenin signaling by decreasing SOST expression promotes the bone anabolic response to mechanical loading.^24,58^ In contrast, deletion of β-catenin from osteocytes/osteoblasts attenuates adaptation to loading.^59^ Here, we showed that SOST expression was decreased in WT mice during tibial loading, which was consistent with the suppressive effect of mechanical stimulus on SOST as reported previously.^60,61^ However, the SOST expression was not suppressed in cKO mice. Correspondingly, the increase of β-catenin expression and osteoblast activity seen in WT mice were ablated in cKO mice. It is worth noting that other inhibitors of the canonical Wnt/β-catenin signaling pathway may act along with SOST in mechanotransduction. Previous studies showed that the level of Dickkopf WNT Signaling Pathway Inhibitor 1 (Dkk1) was affected by SOST^62^ and played a compensatory role in regulating the Wnt signaling and anabolic function of bone to loading in the absence of SOST.^63–65^ In addition, an ion channel called Piezo1 highly expressed in osteocytes participates in the process of skeletal mechanosensation^66,67^ by mediating Ca^2+^ influx.^68^ Since the elevated intracellular Ca^2+^ activated Cx43 hemichannels on the osteocyte surface,^69^ Piezo1 is likely involved in the opening of Cx43 hemichannels. Here, as illustrated in Fig. 8, the results indicate that the role of osteocytic integrin α5 in bone anabolic response to mechanical loading is likely through Cx43 hemichannel and released PGE2, which functions in an autocrine/paracrine manner to reduce SOST in osteocytes and increase β-catenin expression in osteoblasts. As a result, osteoblast activity and endosteal bone formation are enhanced. This study demonstrated the importance of integrin α5 in mediating anabolic response to mechanical loading in bone tissue. Due to the scope and depth of the study, here we reported the data generated from male mice. Previous studies reported sex-related differences in the bone anabolic response to loading.^70,71^ To our knowledge, androgen could inhibit the mechanical sensitivity of bone in male mice,^72^ while activation of estrogen enhances the osteogenic response to mechanical loading in female mice.^24,27,73^ The investigation will be continued with female mice, especially in ovariectomized mice, a relevant model for post-menopausal women with high morbidity of osteopenia and osteoporosis.

In summary, this study unveils a new, physiological role of osteocytic integrin α5 in the anabolic response to mechanical loading on endosteal bone surfaces. Moreover, integrin α5 activated by mechanical stimulation in osteocytes regulates the release of PGE2 via the opening of hemichannels. Integrin α5 deletion impeded loading induced SOST suppression, resulting in inhibition of endosteal bone formation response to mechanical loading. Our study demonstrated a crucial relationship between integrin α5 and Cx43 hemichannels in mediating the bone anabolic response to mechanical loading, and further suggests that integrin α5 could serve as a new potential therapeutic target for bone diseases. Our data support further clinical research strategies including combined treatment of low magnitude load-based exercise with antibody drugs that enhance integrin α5 activity. This could provide specific benefits for osteoporotic or older patients whose bones are less sensitive to mechanical stimulation and cannot bear vigorous exercise regiments.^74,75^ In the future, more in-depth preclinical studies will be carried out to obtain a more deep insight into the role of integrin α5 in mechanical response. When key factors/pathways that mediate the mechanical response are identified, it is expected to establish a potential drug target and develop drug candidates that can help to enhance the mechanical responsiveness and anabolic function of aging patients or patients with physical immobility.

## Materials and methods

### Mouse models

Mice deleted of the integrin α5 gene in osteocytes were generated using the 10-kb *Dmp1* promoter-driven Cre.^76^ Briefly, we bred *α5*^*flx/−*^ mice (provided by Dr. Richard Hynes^21^) with 10-kb *Dmp1-Cre* mice (provided by Dr. Steven Harris^77^) to produce *Dmp1-Cre; α5*^*flx/±*^ mice. *Dmp1-Cre*; *α5*^*flx/+*^ mice were then crossed with *α5*^*flx/−*^ mice to produce *α5*^*flx/flx*^ and *Dmp1-Cre*; *α5*^*flx/flx*^ mice. Finally, *Dmp1-Cre*; *α5*^*flx/flx*^ mice were bred with *α5*^*flx/flx*^ mice to obtain *Dmp1-Cre*; *α5*^*flx/flx*^ conditional knockout (cKO) and *α5*^*flx/flx*^ wild-type (WT) mice (Fig. S7A). The genomic DNA from mouse tail was used for genotype identification by PCR (Fig. S7B) and DNA primer as described.^21,76^ All the mouse lines used herein were generated under C57BL/6 J genetic background. Mice were bred in an animal room at the University of Texas Health Science Center at San Antonio (UTHSCSA). Water and food were freely available. All animal protocols were authorized by the UTHSCSA Institutional Animal Care and Use Committee (IACUC).

### Tibial compliance measurement via strain gauging

The relationship of force and strain (compliance) on each left tibia (R^2^ > 0.99) was established in vivo following previously published protocols.^78,79^ Briefly, 15-week-old WT and cKO male mice were sacrificed and their tibias were exposed. A strain gauge (EA-06-015DJ-120; Measurements Group, Raleigh, NC, USA) was placed onto the anterior-medial surface of tibial diaphysis (at 37% of its entire length from the proximal end, Fig. S8A). Loading ranging from 0 to 9.5 N was carried on the left tibia by a loading machine (LM1, Bose, Framingham, MA, USA). A signal collecting device (MR1-350-127, Vishay Measurements Group, Malvern, PA, USA) received signals from the strain gauge to measure tibial compression-induced strain. As shown in Fig. S8B, WT and integrin α5 cKO mice had similar tibial compliances.

### In vivo mechanical loading on tibial bone

Cyclic axial loading was done on left tibias of 15-week-old cKO and WT male mice using a customized device following previous studies.^20,78,79^ Briefly, after anesthetization, the left tibia was placed in a customized apparatus (Fig. S8C). A loading device (7528-10, Masterflex L/S, Vernon Hills, IL, USA) performed a continuous static preload on tibias at 0.5 N. Tibias were loaded for 1 200 με, 600 cycles (5 min) at 2-Hz frequency, with a square pulse form (Fig. S8D). This strain level evokes an anabolic response at diaphysis 37% distal from the proximal end^24,25^ and did not cause potential knee damage induced by tibial loading in this study (Fig. S3). 2-week mechanical loading was used to observe the bone structural and anabolic response, or 5 consecutive days for immunohistochemistry assays and PGE2 determination. Right tibias acted as contralateral, non-loaded controls.

### BMD determination

The BMD of tibia and whole body were monitored using a DEXA scanner (Lunar PIXImus; GE Medical Systems, Piscataway, NJ, USA) under anesthesia, as previously described.^22^ Briefly, the anesthetized mouse was placed on a specimen tray (Lunar PIXImus) and). The femur was positioned at a 90° angle with the tibia. Tibial and whole-body BMD were monitored once a month.

### Micro-computed tomography

The Micro CT 35 scanner (Scanco Medical AG, Bruttisellen, Switzerland) was used for bone structure scanning under the following parameters: 50 Kvp, 145 µA beam intensity, 800 ms integration time, 0.5 mm aluminum filter, 6 µm isotropic voxel dimension, and 2 048 × 2 048 pixel matrix. The trabecular VOI was positioned 74 slices (0.44 mm) distal to the proximal growth plate with an extension of 108 slices (0.65 mm) from the distal side (Fig. S4). In the 108 slices of interest, an irregular contour along the perimeter of the cortical bone was drawn a few pixels away from the endosteal bounder every 10 slices and interpolated to obtain a 3D VOI containing the majority of the trabecular bone. A threshold of 350/1 000 was used for the trabecular VOI for the analysis. The cortical VOI was positioned 50 slices (0.3 mm) were centered at diaphysis 37% distal from the end of the proximal side (Fig. S4). The VOI matches previous studies where the bone formation after mechanical loading is maximal.^25,26^ The cortex with a threshold of 380/1 000 was selected by automated contouring. The MicroCT Analysis Software was used to analyze the trabecular and cortical structural properties (Scanco Medical AG, Bruttisellen, Switzerland).

### Mechanical testing

Tibias without soft tissues and fibulas excised were prepared in saline-soaked gauze at −80 °C. After the tibias were thawed to room temperature, a mechanical testing device (Mach-1 V500CST, Biomomentum, Laval, Canada) was used to perform the test, as described previously.^20^ Briefly, a tibia was positioned on the loading platform along the medial-lateral direction. The loading parameters are 8 mm span, 0.05 mm/sec, and the data was collected at a 200-Hz collecting frequency. The accurate cross-sectional areas were captured under an Infinity microscope camera (Teledyne Lumenera, Ottawa, Canada), and used to calculate material properties.^80^

### Dynamic bone histomorphometry

Calcein (C0875, Sigma-Aldrich, St. Louis, MO, USA) and alizarin red (A5533, Sigma–Aldrich) were intraperitoneally (IP) injected in mice, as described previously.^20^ Tibias embedded in methyl methacrylate were cut transversely at diaphysis 37% distal from the proximal end using a precision wafering saw (PICO 155, PACE Technologies, Tucson, AZ, USA). The cross-sectional surface was then polished to 80-μm thick using sandpaper of grit P1200 on the PHOENIX 4000 system (BUEHLER, Lake Bluff, IL, USA). Fluorescent labels were imaged by a fluorescence microscope (BZ-X710, KEYENCE, Itasca, IL, USA). Only green, red, or yellow (overlap between green and red) labels on bone surfaces were defined as single labels.^41^ The MS/BS, MAR, and BFR/BS were calculated using the NIH ImageJ, described previously.^20^

### Dye uptake in vivo study

We assessed osteocytic hemichannel activity in tibias in situ for 15-week-old male cKO and WT mice described previously.^17,20^ Briefly, 20 mg·mL^−1^ EB dye was injected into the tail vein. After 10 min loading and 40 min rest, anesthetized mice were under heart perfusion to fix the osteocytes in tibias. Tibias were embedded in the sagittal orientation in optimum cutting temperature compound (OCT), and frozen sagittal sections (12-μm-thickness) were generated. After the nuclei staining with 4’,6-diami- dino-2-phenylindole (DAPI), images were taken with a fluorescence microscope (BZ-X710) for analyzing Evans blue fluorescence intensity in osteocytes. A 2 400 μm long region at diaphysis 37% distal from the proximal end in PL was used for quantification by the NIH ImageJ software (NIH, USA).

### PGE2 measurement

The levels of PGE2 in the serum and tibia bone were quantified using the PGE2 ELISA kit (#514010, Cayman Chemical, Ann Arbor, MI, USA). In this study, four hrs after completion of the five-day tibial loading, whole blood samples were obtained from mouse orbital sinus. Serum was collected following centrifugation at 200 x *g* for 15 min and stored at −80 °C. Tibial diaphysis without bone marrow and soft tissues were homogenized in liquid nitrogen. The concentration of PGE2 in serum and tibias was quantified using the PGE2 ELISA kit and calibrated to total protein concentration determined by a BCA assay.

### Western blotting

Bone marrow-flushed tibias were pulverized in liquid nitrogen and lysed in lysis buffer. The total membrane extract was prepared as previously reported.^49^ Briefly, lysates were centrifuged at 45 000 × *g* for 45 min and re-suspended in lysis buffer with 1% sodium dodecyl sulfate (SDS). The membrane protein was collected and determined concentration using a BCA assay. Proteins on nitrocellulose membranes were detected with an antibody for integrin α5 (1:800 dilution, PA5-82027, Invitrogen, Waltham, MA, USA) or β-actin (1:2 000 dilution, MA515739, Invitrogen, Waltham, MA, USA), and visualized by a Licor Odyssey Infrared Imager (Lincoln, NE, USA). The band intensity was quantified by the NIH ImageJ software (NIH, USA).

### Histomorphometry, TUNEL, Ploton-silver staining, Safranin O staining, and immunohistochemistry

After decalcification, tibias were embedded sagittally in paraffin blocks, and longitudinal sections (5 mm) were obtained. Static bone histomorphometry was analyzed at diaphysis 37% distal from the proximal end in the PL region. Tartrate resistant acid phosphatase (TRAP) was adopted to determine the osteoclast activity as described previously.^22^ Following the staining, the multinucleated (≥3 nuclei) TRAP-positive osteoclasts were quantified on the endosteal surface. Toluidine blue staining was utilized to identify the osteoblasts. H&E staining was adopted to quantify the numbers of empty and total osteocytic lacunae. Ploton-silver staining as described previously^81^ was used for visualization and quantification of the osteocyte lacuno-canalicular network close to endosteal and periosteal surfaces. Safranin-O and Fast-Green staining were used for scoring osteoarthritic damage in MTP and MFC regions following the OARSI guidelines.^82^ The In Situ Cell Death Detection Kit (Roche, Pleasanton, CA, USA) was utilized to detect apoptotic osteocytes at diaphysis 37% distal from the proximal end in the PL region, as described previously.^22^ After antigen retrieval described previously,^20^ sections were probed overnight at 4 °C with an antibody for β-catenin (ab16051, 1:200, Abcam, Waltham, MA, USA), COX-2 (12375-1-AP, 1:200, Proteintech, Rosemont, IL, USA), and sclerostin (SOST) (AF1589, 1:400, R&D systems, Minneapolis, MN, USA), followed with corresponding secondary antibody. The chromogenic reaction was performed with DAB Chromogen. The sections were counter-stained with hematoxylin. Images were captured at diaphysis 37% distal from the proximal end in the PL region using a microscope (BZ-X710) and quantified using the NIH ImageJ software (NIH, USA).

### RT-qPCR

Tibial diaphysis without soft tissues and bone marrow were pulverized in liquid nitrogen. Total RNA was extracted by using TRIzol and synthesized to cDNA by a high-capacity cDNA reverse transcription kit (#4388950, Applied Biosystems, Carlsbad, CA, USA). The ABI 7900 PCR device (Applied Biosystems) and SYBR Green (#1725124, Bio-Rad Laboratories, Hercules, CA, USA) were used to analyze mRNA levels. Housekeeping GAPDH was utilized to calculate the relative gene expression by normalizing it to the control tibia (2^−∆∆Ct^).^83^ Primer sequences used are as listed: *COX-2:* forward: CCTTCTCCAACCTCTCCTACTA; Reverse: GGAAGCTCCTTATTTCCCTTCA. *Sost:* forward: CATCCCAGGGCTTGGAGAGTA; Reverse: TGTCAGGAAGCGGGTGTAGT. *β-catenin:* forward: GACACCTCCCAAGTCCTTTATG; Reverse: CTGAGCCCTAGTCATTGCATAC. *Gapdh*: forward: CTTCAACAGCAACTCCCACTCTTC; Reverse: TCTTACTCCTTGGAGGCCATGT.

### Statistical analysis

IBM SPSS Statistics 24 (SPSS, Chicago, IL, USA) and GraphPad Prism 7 (GraphPad Software, La Jolla, CA, USA) were used to perform statistical analysis, described previously.^20^ Variance homogeneity was evaluated using the Levene test, and normal distribution was determined by the Shapiro–Wilk test. The paired t-test compares the contralateral and loaded tibias within the same genotype. Student unpaired t-test compares WT and cKO mice within loaded or control groups. All data are shown as Means ± SD. *P* < 0.05 indicatess significant.

## Supplementary information

Supplemental figures
