# Supplementary material for: Osteocytes regulate bone anabolic response to mechanical loading in male mice via activation of integrin α5
Source: Bone Res. 2022 Jul 18;10:49. doi: 10.1038/s41413-022-00222-z (PMC9293884; doi:10.1038/s41413-022-00222-z)
Supplement: Supplementary file 1 — Supplemental figures [file 41413_2022_222_MOESM1_ESM.pdf]

Supplemental Figure Legends

Figure S1. Deletion of integrin α5 did not affect the body weight and bone bone mineral density. (A) The rate of body weight gain does not differ between from WT and cKO mice. n=21/group. **(B)** Whole-body or **(C)** tibial bone mineral density (BMD) measured by DEXA did not show any difference in WT and cKO mice. n=21/group. Data are expressed as mean ± SD. Statistical analysis was performed using student unpaired t test.

Figure S2. Deletion of integrin α5 in osteocytes affect osteocyte dendrites. (A, C) Silver-nitrate-stained images of tibial cortical bone from WT and cKO mice showed the osteocyte dendrites (in black patterns) closed to endosteal **(A)** and periosteal surface **(C)** at 37% location from the proximal bone end. Scale bar: 20 µm. **(B, D)** Quantification of the lacuno-canalicular area normalized to bone area in a region closed to periosteal endosteal **(B)** and periosteal surface **(D)**. n=3/group. Data are expressed as mean ± SD. Statistical analysis was performed using student unpaired t test.

Figure S3. Cartilage matrix was not altered after cyclic compressive loading of the knee joint. (A) Safranin O-fast green staining of the medial articular cartilage in both WT and cKO mice. Scale bar, 50 µm. **(B)** Histologic scores of cartilage degeneration revealed no damage to the cartilage matrix following mechanical loading in tibia and femur bone, for both WT and cKO mice. n=4/group. Data are

expressed as mean ± SD. Statistical analysis was performed using paired t test for loaded and contralateral, unloaded tibias or unpaired t test for WT and cKO mice. MTP, medial tibial plateau (above); MFC, medial femoral condyle (below).

Figure S4. Graphical depiction of the VOIs assessed by µCT. A 0.65 mm height VOI starting 0.5 mm below the proximal growth plate was used to assess the trabecular bone. A 0.3 mm height VOI was used to assess cortical bone at the diaphysis 37% distal from the proximal end.

Figure S5. Deletion of integrin α5 in osteocytes reversed the anabolic responses to mechanical loading in tibial metaphyseal trabecular bone. µCT assessment of the metaphyseal trabecular bone for WT and cKO mice: **(A)** trabecular thickness (Tb.Th), **(B)** trabecular number (Tb.N), **(C)** trabecular separation (Tb.Sp), **(D)** bone volume fraction (BV/TV), **(E)** structure model index (SMI) and **(F)** bone mineral density (BMD). n=8/group. **(G)** Representative 3D models of the metaphyseal trabecular bone. Data are expressed as mean ± SD. *, $P<0.05$; **, $P<0.01$. Statistical analysis was performed using paired t test for loaded and contralateral, unloaded tibias or unpaired t test between WT and cKO mice within loaded or control tibias.

Figure S6. Deletion of integrin α5 increased osteoclasts in tibial metaphyseal trabecular bone during mechanical loading. **(A)** Representative images of TRAP-positive osteoclasts (red) in tibial metaphyseal trabecular bone. Scale bar, 80

μm. **(B)** Quantification of TRAP-positive osteoclast number per bone perimeter (N.Oc/BS) and **(C)** osteoclast surface per bone perimeter (Oc.S/BS) in tibial metaphyseal trabecular bone. n=5/group. Data are expressed as mean ± SD. *, $P<0.05$. Statistical analysis was performed using paired t test for loaded and contralateral, unloaded tibias or unpaired t test for WT and cKO mice within a loading condition.

Figure S7. Breeding of integrin α5 deficient mice. (A) Breeding strategy to generate the *α5*$^{flx/flx}$ and osteocyte integrin α5 deficient mice. **(B)** Genomic DNA was purified from tail and PCR was performed for WT integrin α5 allele, "floxed", and 10 kb *Dmp1-Cre*.

Figure S8. Experimental setup for *in vivo* tibia axial compressive loading. (A) A strain gauge was attached to the anterior-medial surface at diaphysis 37% distal from the proximal end. **(B)** The average compliance of the relationship between applied load and resulting strain of WT and cKO mice. n=4/group. **(C)** Diagram of the left tibia positioned at the loading device and the direction of loading. **(D)** Schematic graph of 1 s of the daily 5 min loading signal. Approximately 1200 με was loaded on the 37% VOI. Data are expressed as mean ± SD. Statistical analysis was performed using student unpaired t test at each time point.

Figure S1

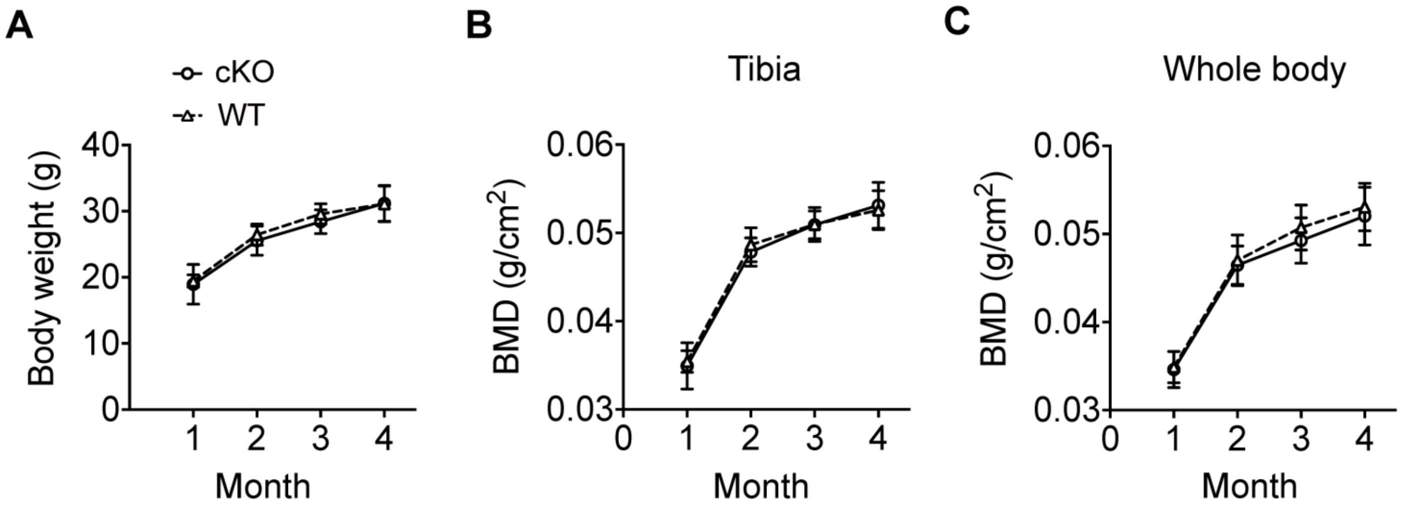
A
B
C
cKO
WT
Tibia
Whole body
Body weight (g)
BMD (g/cm2)
BMD (g/cm2)
40
30
20
10
0
0.06
0.05
0.04
0.03
0
1
2
3
4
Month
Month
Month

Figure S2

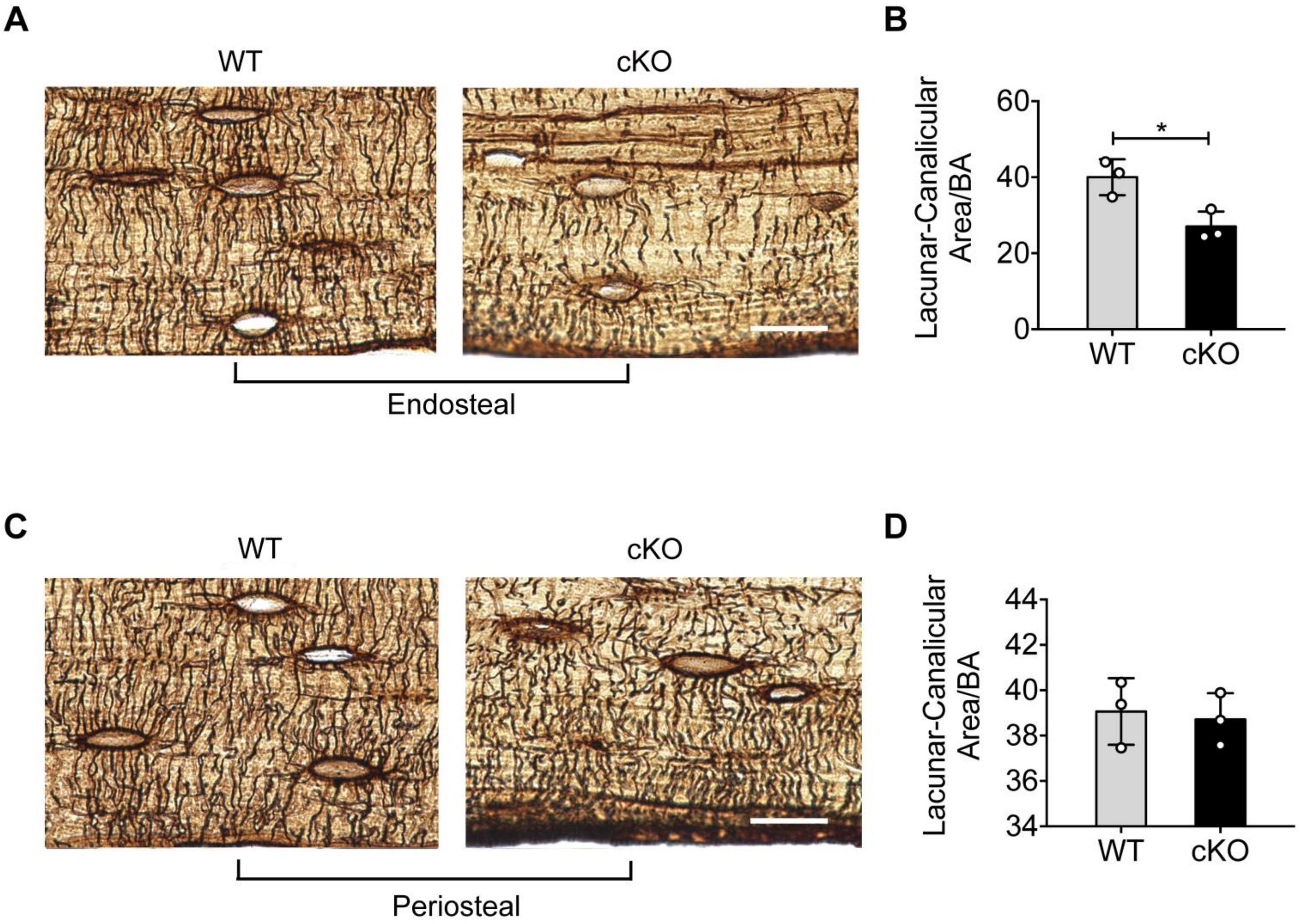
A
WT
cKO
Endosteal
B
Lacunar-Canalicular Area/BA
60
40
20
0
*
WT
cKO
C
WT
cKO
Periosteal
D
Lacunar-Canalicular Area/BA
44
42
40
38
36
34
WT
cKO

Figure S3

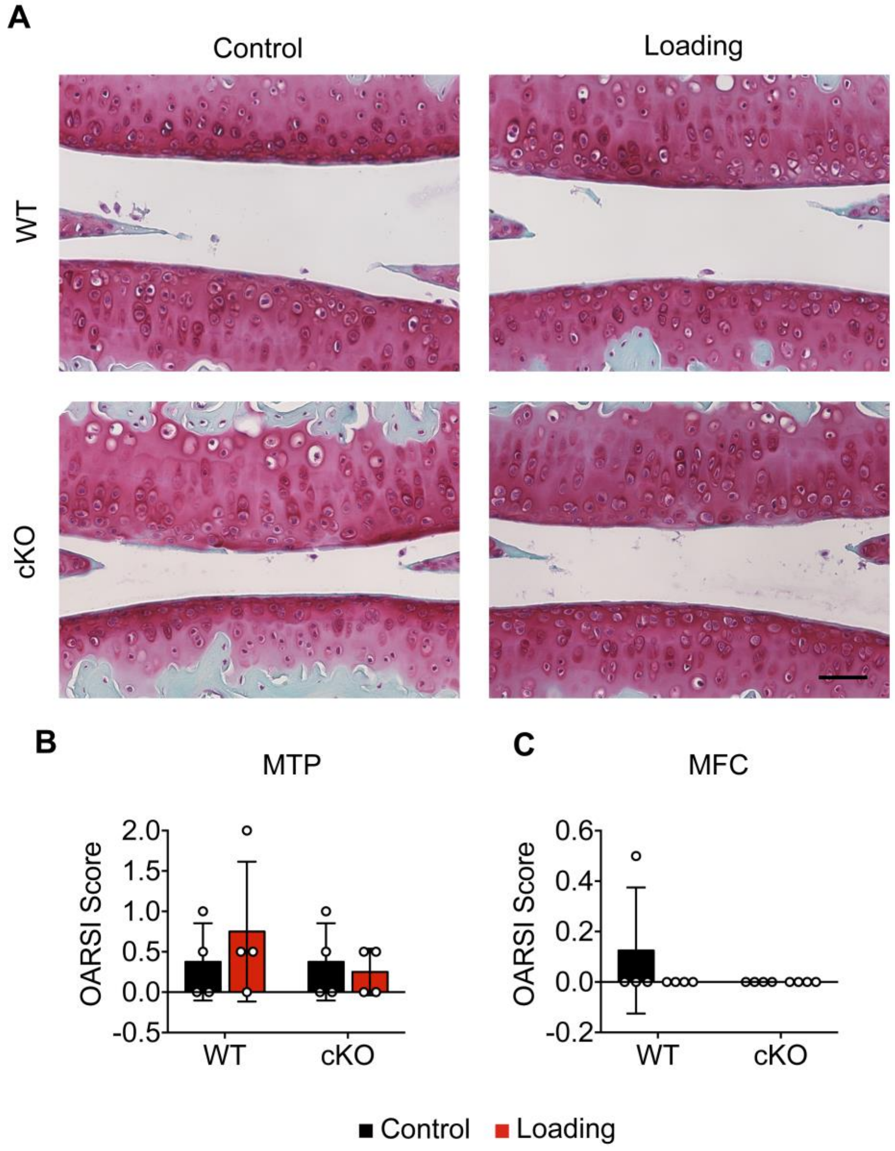

A
Control
Loading
WT
cKO
B
MTP
OARSI Score
2.0
1.5
1.0
0.5
0.0
-0.5
WT
cKO
C
MFC
OARSI Score
0.6
0.4
0.2
0.0
-0.2
WT
cKO
Control
Loading

Figure S4

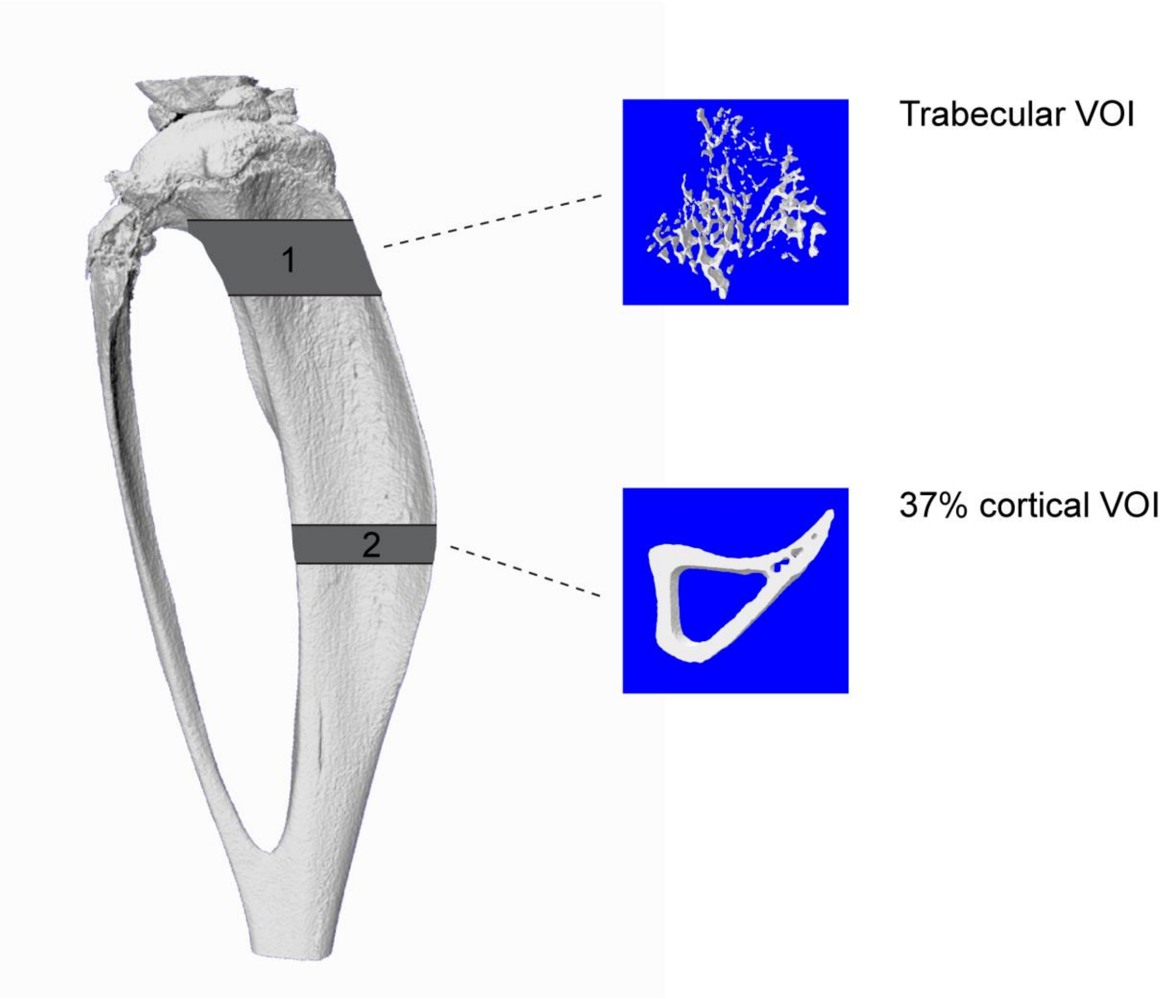

Figure S5

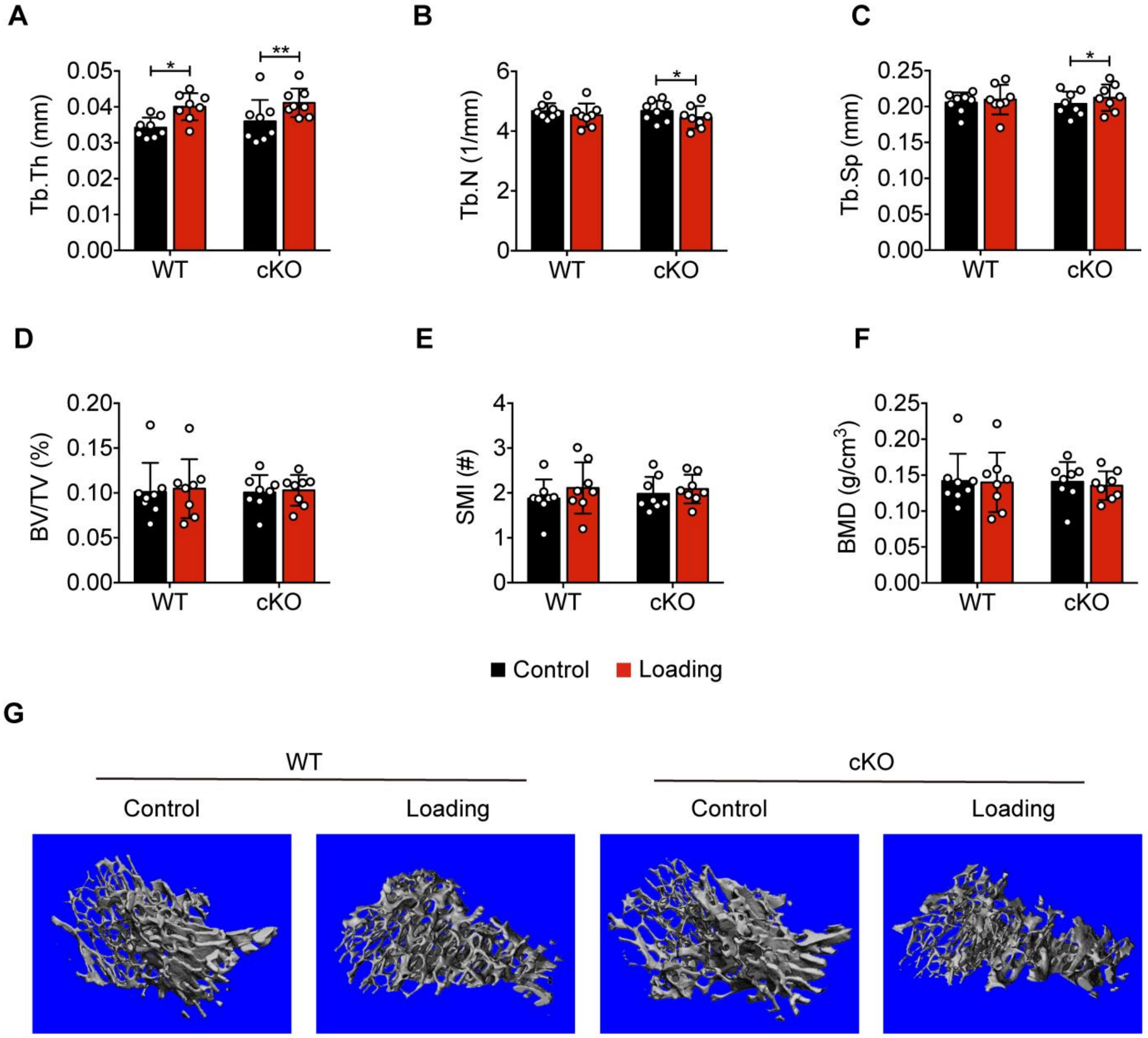

A
B
C
Tb.Th (mm)
Tb.N (1/mm)
Tb.Sp (mm)
D
E
F
BV/TV (%)
SMI (#)
BMD (g/cm3)
WT
cKO
Control
Loading
G
WT
cKO
Control
Loading
Control
Loading

Figure S6

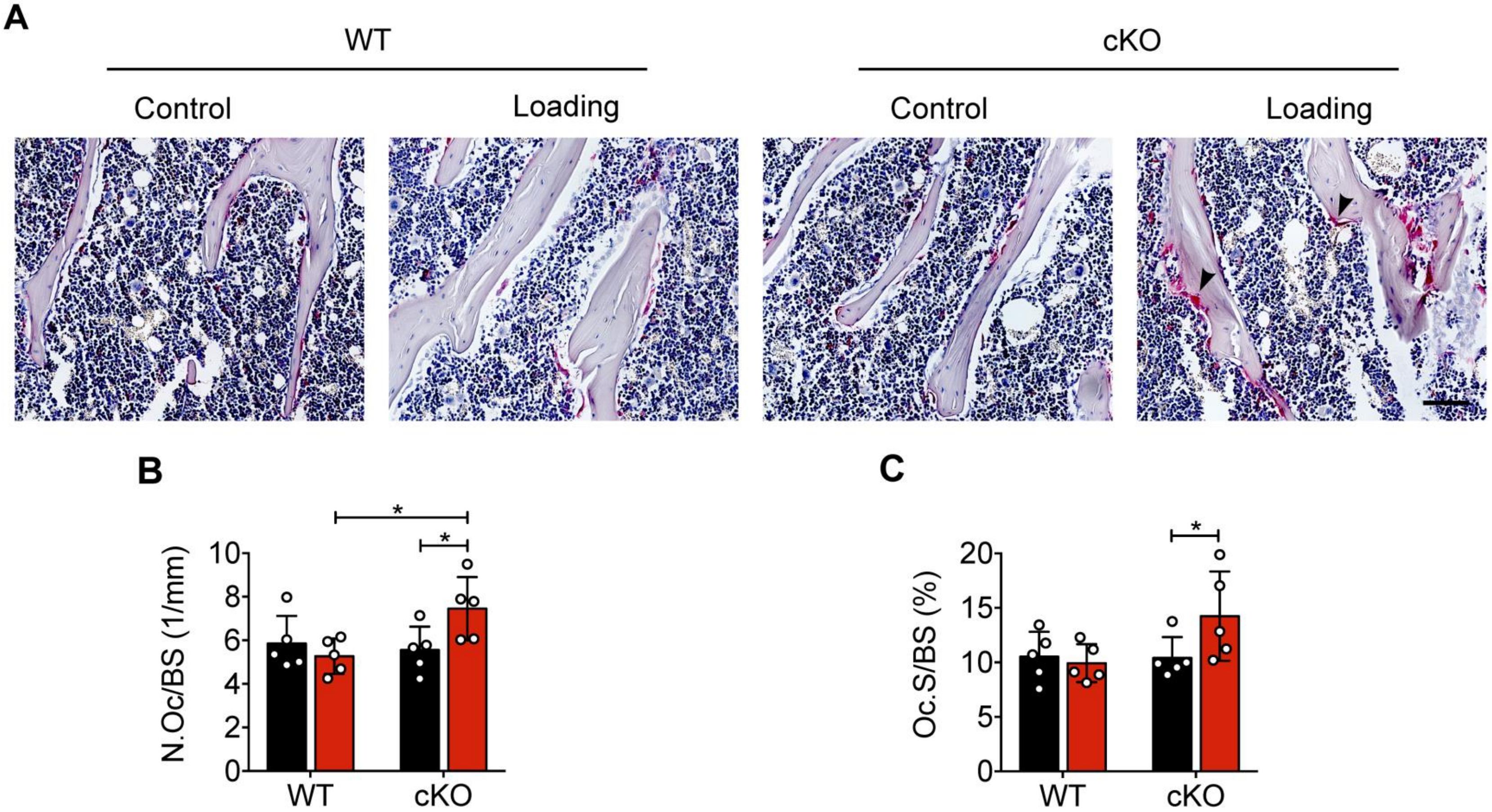
A
WT
cKO
Control
Loading
Control
Loading
B
N.Oc/BS (1/mm)
0
2
4
6
8
10
WT
cKO
*
*
C
Oc.S/BS (%)
0
5
10
15
20
WT
cKO
*

Figure S7

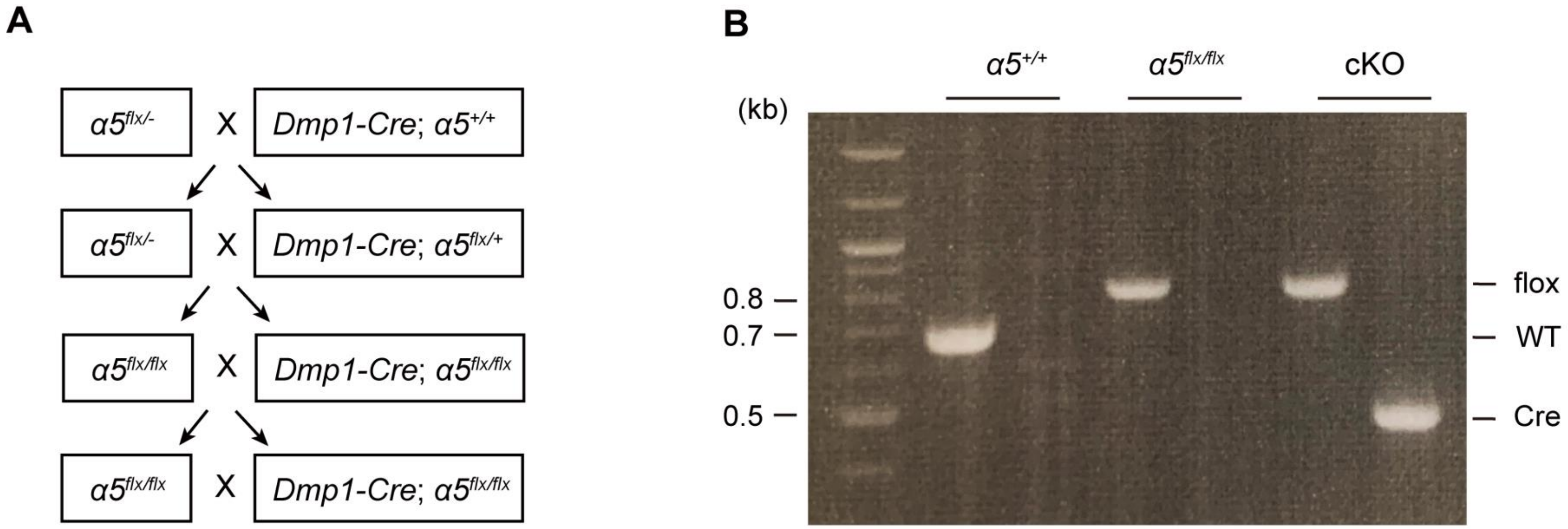
A
α5flx/-
X
Dmp1-Cre; α5+/+
α5flx/-
X
Dmp1-Cre; α5flx/+
α5flx/flx
X
Dmp1-Cre; α5flx/flx
α5flx/flx
X
Dmp1-Cre; α5flx/flx
B
α5+/+
α5flx/flx
cKO
(kb)
0.8
0.7
0.5
flox
WT
Cre

Figure S8

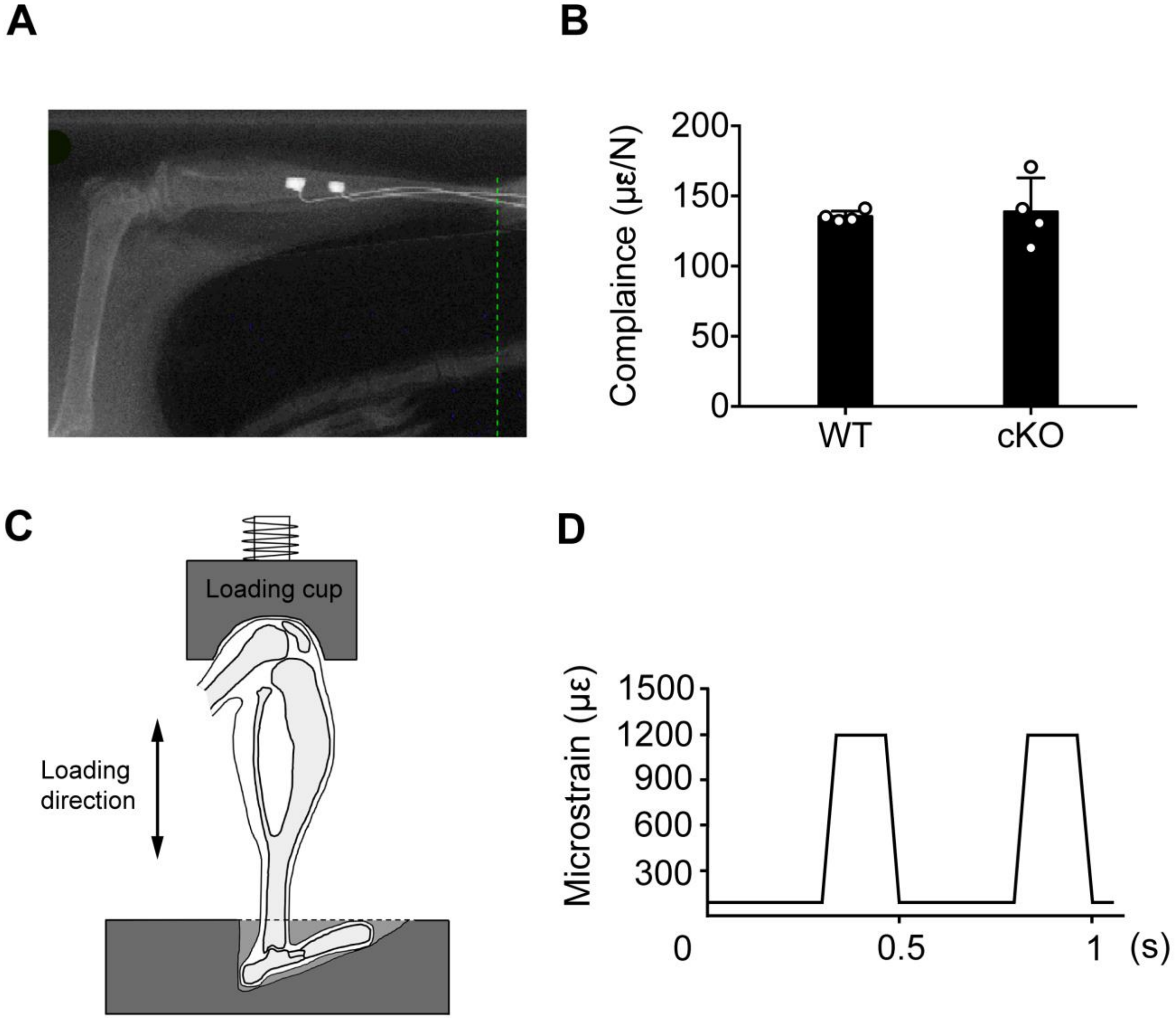
A
B
200
150
100
50
0
Complaince (με/N)
WT
cKO
C
Loading cup
Loading direction
D
1500
1200
900
600
300
Microstrain (με)
0
0.5
1
(s)
